# Serum Exosomes Derived from Irritable Bowel Syndrome Patient Increase Cell Permeability via Regulating miR-148b-5p/RGS2 Signaling in Human Colonic Epithelium Cells

**DOI:** 10.1155/2021/6655900

**Published:** 2021-06-14

**Authors:** Ying Xing, Shan Xue, Jing Wu, Jianhong Zhou, Fangfang Xing, Tianxing Li, Xiaohu Nie

**Affiliations:** ^1^Department of Gastroenterology, 72nd Group Army Hospital, Huzhou University, Huzhou 313000, Zhejiang, China; ^2^Huzhou Central Hospital, Affiliated Central Hospital of Huzhou University, Huzhou 313000, Zhejiang, China

## Abstract

**Aim:**

Irritable bowel syndrome (IBS) is a multifactorial functional bowel disorder characterized by disruption of the intestinal barrier. Circulating exosomal microRNAs (miRNAs) are involved in regulating epithelial barrier function, and upregulation of miR-148b-5p has been detected in IBS. However, whether exosomal miR-148-5p is involved in the IBS pathogenesis remains unclear. This study was aimed at investigating the relationship of exosomal miR-148-5p with colonic epithelial permeability.

**Methods:**

Exosomes were isolated from the serum of IBS patients and healthy controls. HT-29 cells were cultured with the IBS-derived serum exosomes (IBS-exo). Exosome uptake assay was used to evaluate whether the IBS-exo could be absorbed by HT-29 cells. FITC-Dextran flux and transepithelial/endothelial electrical resistance were measured to evaluate epithelial permeability. A luciferase reporter assay was used to determine whether the regulator of G protein signaling- (RGS-) 2 is a target gene of miR-148b-5p.

**Results:**

miR-148b-5p was obviously elevated in the IBS-exo compared to the control-exo. Upregulation of miR-148b-5p was observed in the HT-29 cells cultured with IBS-exo. Exposure to IBS-exo increased cell permeability and decreased RGS2 expression. The IBS-exo-induced alterations were obviously reversed by interfering with the miR-148b-5p expression. Mimicking the IBS-exo treatment, miR-148b-5p overexpression increased cell permeability and downregulated RGS2 expression, which were abrogated by overexpressing RGS2. The luciferase reporter assay revealed that RGS2 was a direct target of miR-148b-5p.

**Conclusions:**

Serum-derived exosomes from IBS patients increase colonic epithelial permeability via miR-148b-5p/RGS2 signaling.

## 1. Introduction

Irritable bowel syndrome (IBS) is a multifactorial functional bowel disorder characterized by abdominal pain, diarrhea, and deregulated bowel habits, affecting the quality of life of 9-23% of the general population [1, 2]. Intestinal barrier is a physical barrier against luminal inflammatory molecules, and intestinal hyperpermeability results in immune activation and chronic low-grade mucosal inflammation [3, 4]. Intestinal hyperpermeability is a critical mechanism responsible for diarrhea and visceral hypersensitivity (VH) of IBS patients [5, 6]. However, the pathophysiology of IBS has not been well understood. MicroRNAs (miRNAs) are crucial for maintaining intestinal epithelial barrier function because of their involvement in modulating expression of tight junction- (TJ-) related proteins. miR-16 and miR-125b participate in modulating claudin-2 and cingulin expression in the jejunum of the patients with diarrhea-predominant IBS, thus impairing epithelial barrier function [7]. *In vivo* experiments show that miR-144 is upregulated and could increase intestinal hyperpermeability through targeting occludin and zonula occludens 1 in IBS rat models [8]. Besides, myeloid-derived miR-223 suppresses intestinal inflammation through decreasing NLRP3 inflammasomes [9]. In IBS, a recent study reported that miR-148-5p is upregulated in the colon mucosa of patients [10]. However, the molecular mechanism of miR-148-5p in IBS has not been fully understood.

Molecular composition of extracellular vesicles (EVs) is a reflector of the physiological status of the producing cells [11]. Blood exosomes are a subgroup of circulating EVs, acting as a transmitter of information and a transporter of protein and RNA from cell to cell or organ [12]. Circulating exosomal miRNAs have attracted increasing attention as potential biomarkers for gastrointestinal cancer in recent years [13]. EVs participate in regulating epithelial barrier integrity and function by affecting the assembly of intestinal epithelial cell functional complexes and modulate trafficking and activity of immune cells by regulating cytokine expression and transport diverse chemokines and lipids [14]. For example, upregulation of miR-29a in the blood exosomes of IBS patients is involved in the regulation of intestinal permeability [15]. Regulators of G protein signaling (RGS) proteins play a role in gastrointestinal inflammation and visceral pain by regulating the G protein-coupled receptors (GPCRs) in the gastrointestinal tract, thereby modulating the activity of endogenous opioid, cannabinoid, and serotonin systems [16]. Moreover, a recent study reports that mesenchymal stem cell-derived exosomal miR-148b-5p mediates the immunosuppressive effect through downregulating 15-LOX-1 in macrophages in inflammatory bowel disease [17]. However, the relationship of miR-148b-5p with intestinal permeability in IBS and the underlying mechanisms remain unclear.

In this study, human colonic epithelium HT-29 cells were cultured with the serum-derived exosomes from IBS patients to measure the alterations of cell permeability. Transfection with miR-148b-5p inhibitor or mimic was used to achieve miR-148b-5p knockdown or overexpression in HT-29 cells.

## 2. Methods

### 2.1. Collection of Human Blood Samples

This study enrolled 20 IBS patients and 20 healthy subjects from Huzhou Center Hospital, Affiliated Center Hospital Huzhou University. A blood sample was collected from each IBS patient or healthy control subject. This study was approved by the ethical committee of Huzhou Center Hospital, Affiliated Center Hospital Huzhou University, and is in compliance with the 1964 Helsinki declaration and its later amendments or comparable ethical standards.

### 2.2. Cell Culture

Human colonic epithelium HT-29 cells were obtained from the cell bank of the Shanghai Biology Institute (Shanghai, China) and cultured in DMEM (Trueline, Kaukauna, WI, USA) medium supplemented with 10% FBS (Thermo Scientific), 2 mM l-glutamine, and 1% P/S (Solarbio, Beijing, China) at 37°C, 5% CO_2_.

### 2.3. Cell Transfection

miR-148b-5p inhibitor for interfering with miR-148b-5p; miR-148b-5p mimic for overexpressing miR-148b-5p, or miNC; and oeRGS2 plasmid for RGS2 overexpression, or oeNC, were transfected into HT-29 cells using Lipofectamine 2000 (Invitrogen, Carlsbad, USA) according to the manufacturer's instructions. After 24 h, the cells were then cultured with serum-derived exosomes from IBS patients or not.

### 2.4. Quantitative Real-Time PCR

Briefly, total RNA was extracted from different samples using a TRIzol Reagent (Invitrogen, Waltham, USA) and reverse transcribed into cDNA using a cDNA synthesis kit (Thermo Scientific, Waltham, USA). The primers used in this study were listed as follows: hsa-miR-148b-5p, F: 5′-CGCGCGAAGT TCTGTTATACAC-3′, R: 5′-AGTGCAGGGTCCGAGGTATT-3′; U6, F: 5′-CTCGCTTCGGCAGCACA-3′, R: 5′-AACGCTTCACGAATTTGCGT-3′; RGS2, F: 5′-GATTGGAAGACCCGTTTG-3′, R: 5′-CCCTGAATGCAGCAAGAC-3′; and GAPDH, F: 5′-GGATTGTCTGGCAGTAGCC-3′, R: 5′-ATTGTGAAAGGCAGGGAG-3′. The relative expression was calculated using the 2 − *ΔΔ*Ct method [18]. GAPDH was used as the internal reference to normalize the expression levels. Each experiment was performed in triplicate.

### 2.5. Western Blot

Briefly, total protein (25 *μ*g) was fractionated on SDS-PAGE gels and subsequently electroblotted to PVDF membranes. The membranes were incubated with primary antibodies overnight (4°C), followed by anti-mouse secondary antibody IgG (Beyotime, Shanghai, China) for 1 h at 37°C. All primary antibodies used in this study were provided as follows: CD9 (Ab92726, Abcam, UK), CD63 (Ab134045, Abcam, UK), RGS2 (Ab36561, Abcam, UK), and GAPDH (60004-1-1G, Proteintech, USA). The experiment was conducted in triplicate.

### 2.6. FITC-Dextran Flux and Transepithelial/Endothelial Electrical Resistance (TEER)

For the measurement of the dextran flux, briefly, cells were inoculated into the upper chamber of a 24-well Transwell plate and cultured in the medium added with fluorescently labeled dextran (FITC-Dextran, 1 mg/mL) for 24 h. Intensity of FITC fluorescence at 490 nm was measured using a microplate reader (Pulangxin, Beijing, China), and the permeability rate was calculated.

Millicell ERS-2 (Millicell ERS-2, Millipore, USA) was used to examine TEER values. The TEER value was calculated as follows: TEER value (*Ω* · cm^2^) = TEER (*Ω*) × surface area (0.6 cm^2^).

### 2.7. Exosome Isolation and Observation

Exosomes were precipitated by using an exosome precipitation solution (Exo-Quick; System Biosciences) following the manufacturer's instructions [19]. Ultrastructure and size distribution of the purified exosomes were analyzed by transmission electron microscopy (TEM) and NanoSight (Malvern), respectively. Protein markers, CD9 (Ab92726, Abcam, UK) and CD63 (Ab134045, Abcam, UK), were determined by immune blotting.

### 2.8. Exosome Uptake Assay

To determine whether HT-29 cells could uptake IBS-exos, we stained exosomes with PKH67 (Sigma, USA) according to standard protocols as previously reported (2). We then cocultured these labeled exosomes with HT-29 cells. Finally, we stained HT-29 cells with DAPI (Sigma, USA). After 24 hours, the cells were observed with confocal microscopy.

### 2.9. Luciferase Reporter Assay

The HT-29 cells cultured in 24-well plates were transfected with a luciferase reporter vector carrying wild type (WT) or mutant (Mut) 3′UTR of RGS2 for miR-148b-5p. The luciferase activity was evaluated using a Dual-Luciferase Reporter Assay System (Promega, Madison, USA).

### 2.10. Statistical Analysis

GraphPad Prism software (version 7.0, La Jolla, CA, USA) was applied for statistical analyses. Triplicate replications were necessary for each experiment. Data were displayed as mean ± SD. Comparison between two groups was performed using Students' *t* test, while comparison among multiple groups was performed using one-way analysis of variance. Statistical significance was defined using *p* value < 0.05.

## 3. Results

### 3.1. miR-148b-5p Was Upregulated in the Serum of IBS Patients

**Figure 1:**
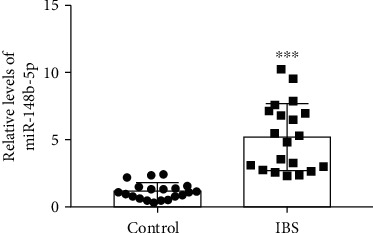
miR-148b-5p is upregulated in the serum-derived exosomes of patients with irritable bowel syndrome compared to the healthy control. *N* = 20 for each group. ^∗∗∗^*p* value < 0.001 vs. Control.

The serum level of miR-148b-5p was assayed in the IBS patients (*N* = 20) and the healthy subjects (*N* = 20). Compared with the healthy controls, the IBS patients had a significantly higher level of miR-148b-5p in serum (*p* value < 0.001, Figure 1).

### 3.2. Serum Exosomes Derived from IBS Patients Induced an Upregulation of miR-148b-5p in HT-29 Cells

**Figure 2:**
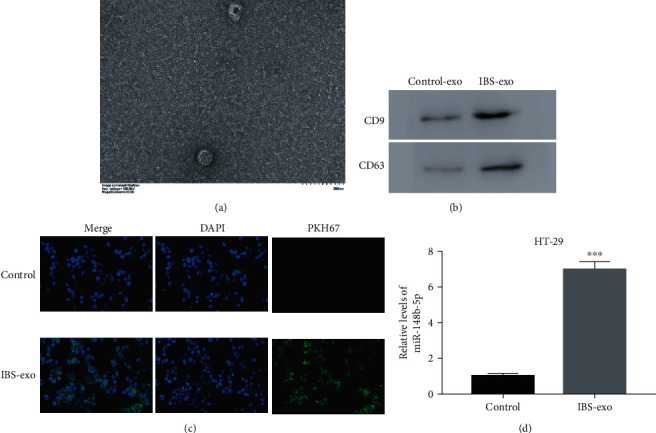
Serum exosomes derived from IBS patients (IBS-exos) induce miR-148b-5p upregulation in HT-29 cells. (a) The morphology of serum exosomes derived from IBS patients. (b) Both CD9 and CD63 are positive for IBS-exos and the corresponding control-exos. (c) The PKH67-labelled (green) immature IBS-exos are cocultured with HT-29 cells. Then, the HT-29 cells are fixed and stained with DAPI (blue). The uptake of IBS-exos by HT-29 cells is observed under a confocal microscope. (d) The relative mRNA level of miR-148b-5p is upregulated in the HT-29 cells cocultured with IBS-exo. ^∗∗∗^*p* value < 0.001 vs. Control.

Serum exosomes were isolated from IBS patients (IBS-exo) and healthy controls (control-exo) and identified by surface markers CD9 and CD63. Under TEM, exosomes were round in shape (Figure 2(a)). A positive expression of CD9 and CD63 was found in IBS-exos and control-exo (Figure 2(b)). This result suggested exosomes were successfully isolated from the IBS patients and the healthy controls. The isolated exosomes from IBS patients were then labeled with PKH67 and cocultured with HT-29 cells. As shown in Figure 2(c), the PKH67-labeled IBS-exo could be internalized by HT-29 cells. Besides, the HT-29 cells cocultured with IBS-exo had a remarkably elevated level of miR-148b-5p than the HT-29 cells cocultured with control-exos (*p* value < 0.001, Figure 2(d)).

### 3.3. miR-148b-5p Inhibitor Suppressed the Effects of IBS-Exo on Cell Permeability and RGS2 in HT-29 Cells

**Figure 3:**
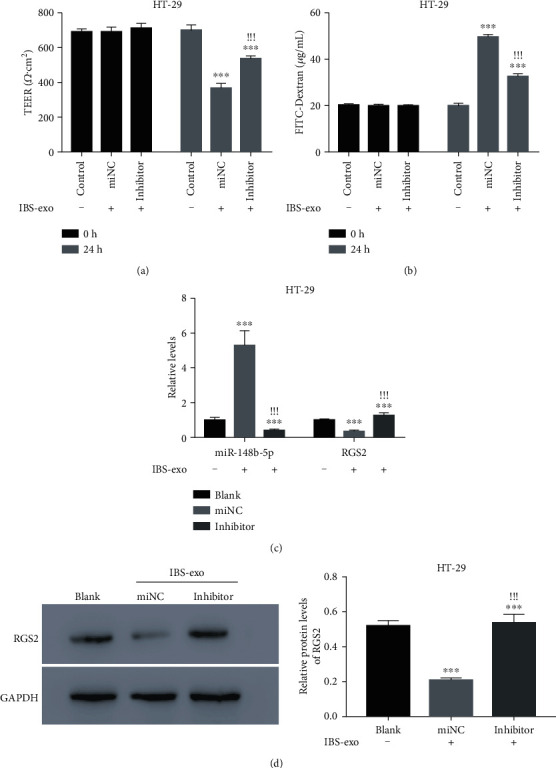
miR-148b-5p inhibitor suppresses the effects of IBS-exo on cell permeability and RGS2 in HT-29 cells. (a) TEER value of the IBS-exo-cultured HT-29 cells transfected with miR-148b-5p inhibitor or miNC. (b) FITC-Dextran influx of the IBS-exo-cultured HT-29 cells transfected with miR-148b-5p inhibitor or miNC. (c) Quantitative PCR is used to examine miR-148b-5p and RGS2 mRNA in HT-29 cells with different treatments as indicated above. (d) RGS2 protein level is detected in HT-29 cells with different treatments. Prior to IBS-exo treatment for 24 h, HT-29 cells are pretransfected with miR-148b-5p inhibitor or miNC. ^∗∗∗^*p* value < 0.001 vs. control, ^!!!^*p* value < 0.001 vs. miNC.

Cell permeability was evaluated using measurement of the FITC-Dextran flux and TEER. As shown in Figures 3(a) and 3(b), the HT-29 cells that were cocultured with IBS-exo had increased cell permeability, as suggested by significantly reduced TEER and increased FITC-Dextran, compared to the control cells (*p* value < 0.001). To investigate the role of miR-148b-5p in the molecular mechanisms behind the effect of IBS-exo on the permeability of HT-29 cells, the cells were pretransfected with the miR-148b-5p inhibitor for 24 h to silence miR-148b-5p before a 24 h coculture with IBS-exo. The miR-148b-5p inhibitor successfully downregulated miR-148b-5p in HT-29 cells (*p* value < 0.001, Figure S1). Pretransfection of the miR-148b-5p inhibitor significantly restored the permeability of the cells cocultured with IBS-exo (Figures 3(a) and 3(b)). Moreover, the coculture with IBS-exo significantly upregulated miR-148b-5p, which was reversed by transfection with the miR-148b-5p inhibitor (*p* value < 0.001, Figure 3(c)) These findings suggest that IBS-exo increases the permeability of HT-29 cells partly via upregulating miR-148b-5p.

Using TargetScan, we found that RGS2 was a predicted target of miR-148b-5p. Noticeably, IBS-exo treatment caused an obvious downregulation of RGS2 at mRNA and protein levels in HT-29 cells, which was abrogated by transfection with the miR-148b-5p inhibitor (*p* value < 0.001, Figures 3(c) and 3(d)). Based on these observations, we hypothesize that RGS2 is a potential target of miR-148b-5p.

### 3.4. miR-148b-5p Regulated RGS2 Expression through Binding on Its 3′UTR

**Figure 4:**
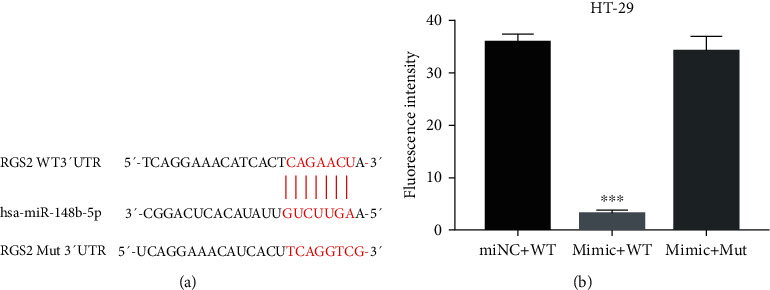
miR-148b-5p inhibits transcription of RGS2 through binding to its 3′UTR. (a) Wild type (WT) and mutant (Mut) binding sites in the 3′UTR of RGS2 for miR-148b-5p. (b) Luciferase activity of the HT-29 cells with different treatments. HT-29 cells are cotransfected with miR-148b-5p mimic and the luciferase reporter vector containing WT or Mut binding sites. ^∗∗∗^*p* value < 0.001 vs. miNC+WT.

To determine whether RGS2 was a downstream target of miR-148b-5p, the luciferase reporter vector containing wide type (WT) or mutant (Mut) binding sites was cotransfected with the miR-148b-5p mimic into HT-29 cells (Figure 4(a)). miR-148b-5p overexpression markedly reduced the luciferase activity in the cells cotransfected with WT 3′UTR of RGS2 (*p* value < 0.001) but induced little change in the luciferase activity in the cells cotransfected with the mutant 3′UTR of RGS2 (Figure 4(b)). It implies that the 3′UTR of RGS2 is a target of miR-148b-5p.

### 3.5. RGS2 Overexpression Abolished the Effects of miR-148b-5p Overexpression on Permeability of HT-29 Cells

**Figure 5:**
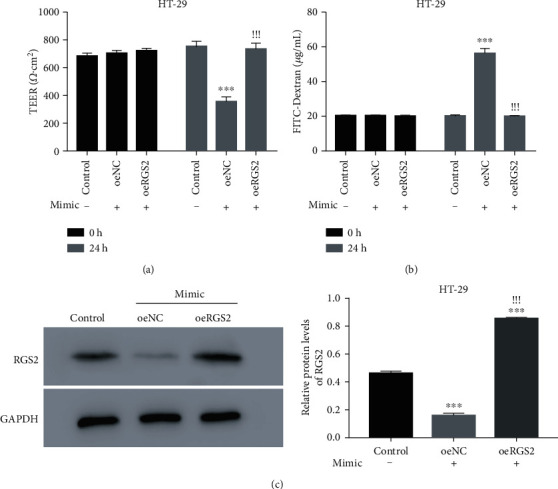
RGS2 overexpression disrupts the effects of miR-148b-5p overexpression on permeability of HT-29 cells. (a) TEER value of the HT-29 cells cotransfected with miR-148b-5p mimic and oeRGS2 or oeNC. (b) FITC-Dextran influx of the HT-29 cells cotransfected with miR-148b-5p mimic and oeRGS2 or oeNC. (c) RGS2 protein levels of the HT-29 cells with different treatments. HT-29 cells are cotransfected with miR-148b-5p mimic and oeRGS2 or oeNC. ^∗∗∗^*p* value < 0.001 vs. control, ^!!!^*p* value < 0.001 vs. oeNC.

We further explored whether RGS2 was involved in miR-148b-5p promoting colonic epithelial permeability. miR-148b-5p or RGS2 was overexpressed in HT-29 cells via the miR-148b-5p mimic or oeRGS2 transfection (Figures S1 and S2). Overexpressing miR-148b-5p reduced the TEER value and increased the FITC-Dextran influx, similar to the changes induced by IBS-exo treatment, suggesting that the miR-148b-5p overexpression increased cell permeability (*p* value < 0.001, Figures 5(a) and 5(b)). The increased cell permeability was restored by transfection with oeRGS2 (Figures 5(a) and 5(b)). Besides, RGS2 expression was attenuated as a result of miR-148b-5p overexpression but was augmented in response to cooverexpression of RGS2 and miR-148b-5p (Figure 5(c)). These findings imply that miR-148b-5p may regulate colon epithelial permeability via targeting RGS2.

## 4. Discussion

EVs are present in various biological fluids and implicated in diverse pathological processes [11]. Studies on functions and mechanisms of exosomes in IBS would help us to gain more insights into the molecular characteristics of IBS and lay a base for identification of more diagnostic and therapeutic biomarkers of clinical significance [14]. Molecular composition of EVs is a reflector of the physiological status of the producing cells [20]. It has been reported that the EVs isolated from inflammatory bowel disease patients activate colonic epithelial cells and macrophages [21]. In the current study, we isolated and purified serum exosomes from IBS patients and treated human colonic epithelium HT-29 cells with these exosomes to establish an *in vitro* model of IBS. Our study provided convincing evidence that the serum-derived exosomes from IBS patients were uptaken by human colonic epithelial cells. Moreover, the HT-29 cells cultured with IBS-exo showed decreased TEER and increased FITC-Dextran flux, suggesting that IBS-exo increases colonic epithelial permeability. These observations reveal that IBS-exo plays a role in the disruption of intestinal barrier function in IBS. Consistently, emerging studies have demonstrated that EVs are big players in intestinal epithelial integrity [22, 23].

Liquid biopsy often represents a rapid and noninvasive alternative to tissue biopsy. Additionally, it allows for the longitudinal evaluation of cancer evolution [24, 25]. A blood-based liquid biopsy consists of the isolation and analysis of disease-derived or disease-associated components that circulate in the bloodstream, such as microRNA (miRNA) and noncoding RNAs (ncRNAs) [26]. In the present study, we collected the blood samples of IBS patients and healthy controls. Our results have indicated that miR-148b-5p was upregulated in the blood of IBS patients. These findings demonstrated that miR-148b-5p has the potential value as a biomarker for IBS clinic practice and can be used as a target for IBS treatment.

The miRNA-148/-152 family (miR-148a, miR-148b and miR-152) is involved in regulating differentiation and function of immune cells and presents candidate therapeutic targets for chronic inflammatory intestinal diseases [27]. Upregulation of miR-148-5p is found in colon mucosa of IBS patients [10]. In accordance with previous findings, the current study showed that miR-148b-5p was dramatically upregulated in IBS-exo as well as in the HT-29 cells cultured with IBS-exo. Moreover, the present study showed that IBS-exo promoted cell permeability, which was partly reversed by miR-148b-5p downregulation. Furthermore, miR-148b-5p overexpression increased epithelial permeability, similar to IBS-exo treatment. These results indicate that IBS-exo increases colon epithelial permeability partly by deliveringmiR-148b-5p. This is the first study, to the best of our knowledge, characterizing the regulatory role of miR-148b-5p in colon epithelial permeability in IBS. It has been established that miRNAs manipulate the expression of tight junction proteins critical for preserving epithelial barrier integrity [28]. miR-148b-5p may impact colon epithelial permeability in IBS by manipulating the expression of tight junction proteins.

RGS proteins participate in modulating GPCRs that are involved in regulating intestinal inflammation and visceral pain [16]. RGS2 belongs to the R4/B subfamily and negatively regulates G protein signaling that activates GTPase [29]. Using TargetScan, we predicted that RGS2 was a promising target of miR-148b-5p. Our results suggest that RGS2 expression is regulated by miR-148b-5p. Moreover, miR-148b-5p binds to 3′UTR of RGS2 to modulate its expression. This is the first report, as far as we know, suggesting that miR-148b-5p is a negative modulator of RGS2 expression. Furthermore, our study showed that similar to IBS-exo treatment, miR-148b-5p overexpression promoted colon epithelial permeability and was reversed by RGS2 overexpression. It implies that miR-148b-5p affects colonic epithelial permeability by regulating RGS2 expression. The miR-148b-5p/RGS2 signaling pathway may be a critical mechanism underlying the impairing effect of IBS-exo on the colonic epithelial barrier. It has been documented that increased permeability of the intestinal barrier causes microbial translocation and contributes to VH and exacerbation of intestinal inflammation [30–32]. It has been reported that RGS2 negatively modulates signaling of *κ*-opioid receptors associated with pain perception and intestinal motility [33]. RGS2 protects against airway inflammation and increases the expression of proinflammatory S100A12 in airway epithelial cells [34, 35]. We therefore infer that miR-148b-5p increases colonic epithelial permeability by downregulating RGS2, thereby intensifying inflammation and VH in the pathogenesis of IBS. Our study supports the pursuit of miR-148b-5p/RGS2 signaling as a promising target for IBS. Future efforts dedicated to define the detailed mechanisms of miR-148b-5p/RGS2 signaling the pathophysiology of IBS are needed.

## 5. Conclusion

In summary, we suggest that serum-derived exosomes from IBS patients promote colonic epithelial permeability by upregulating miR-148b-5p to suppress RGS2 expression in colonic epithelial cells. This study opens future avenues for identifying novel therapeutic targets to treat IBS and other associated bowel diseases.

## Data Availability

The datasets used and/or analyzed during the current study are available from the corresponding author on reasonable request.

## Supplementary Materials

Supplementary Materials Figure S1: miR-148b-5p is silenced or overexpressed by transfecting with miR-148b-5p inhibitor or mimic. ^∗∗∗^*p* value < 0.001 vs. miNC. Figure S2: RGS2 overexpression is induced in HT-29 cells. mRNA (A) and protein levels (B) of RGS2 in the HT-29 cells transfected with oeNC or oeRGS2. ^∗∗∗^*p* value < 0.001 vs. oeNC.
