# Supplementary material for: Serum Exosomes Derived from Irritable Bowel Syndrome Patient Increase Cell Permeability via Regulating miR-148b-5p/RGS2 Signaling in Human Colonic Epithelium Cells
Source: Gastroenterol Res Pract. 2021 Jun 14;2021:6655900. doi: 10.1155/2021/6655900 (PMC8219443; doi:10.1155/2021/6655900)
Supplement: Supplementary Materials — Figure S1: miR-148b-5p is silenced or overexpressed by transfecting with miR-148b-5p inhibitor or mimic. ∗∗∗p value < 0.001 vs. miNC. Figure S2: RGS2 overexpression is induced in HT-29 cells. mRNA (A) and protein levels (B) of RGS2 in the HT-29 cells transfected with oeNC or oeRGS2. ∗∗∗p value < 0.001 vs. oeNC. [file 6655900.f1.docx]

Supplementary Materials:

Figure S1: miR-148b-5p is silenced or overexpressed by transfecting with miR-148b-5p inhibitor or mimic. *** p-value < 0.001 vs. miNC.

Figure S2: RGS2 overexpression is induced in HT-29 cells. mRNA (A) and protein levels (B) of RGS2 in the HT-29 cells transfected with oeNC or oeRGS2. *** p-value < 0.001 vs. oeNC.
